# Research and Implementation of Vehicle Target Detection and Information Recognition Technology Based on NI myRIO

**DOI:** 10.3390/s20061765

**Published:** 2020-03-22

**Authors:** Hongliang Wang, Shuang He, Jiashan Yu, Luyao Wang, Tao Liu

**Affiliations:** National Key Laboratory for Electronic Measurement Technology, Key Laboratory of Instrumentation Science & Dynamic Measurement (North University of China), Ministry of Education, North University of China, Taiyuan 030051, China; 1306024217@st.nuc.edu.cn (S.H.); 1506024121@st.nuc.edu.cn (J.Y.); 1205014127@st.nuc.edu.cn (L.W.); 1201014310@st.nuc.edu.cn (T.L.)

**Keywords:** vehicle target detection, vehicle information recognition, NI myRIO

## Abstract

A vehicle target detection and information extraction scheme based on NI (National Instruments) myRIO is designed in this paper. The vehicle information acquisition and processing method based on image recognition is used to design a complete vehicle detection and information extraction system. In the LabVIEW programming environment, the edge detection method is used to realize the vehicle target detection, the pattern matching method is used to realize the vehicle logo recognition, and the Optical Character Recognition (OCR) character recognition algorithm is used to realize the vehicle license plate recognition. The feasibility of the design scheme in this paper is verified through the actual test and analysis. The scheme is intuitive and efficient, with the high recognition accuracy.

## 1. Introduction

In the current intelligent transportation system (ITS), the accuracy and the efficiency of the real-time vehicle targets detection and vehicle identity recognition become more important with the increasing number of vehicles and the increasingly complex traffic environment [1,2]. Traffic information monitoring technology based on image recognition has become a hot spot in the transportation field in recent years. It collects traffic video by the camera, and uses image recognition and processing technology to identify and process the target area, monitor the traffic condition, and extract traffic information through a certain algorithm, which has the advantages of convenient installation, wide coverage, and high detection accuracy. With the development of computer technology, traffic information detection technology based on image recognition and processing technology has gradually matured and improved, and will be more and more widely used in intelligent transportation systems [3].

Vehicle detection and recognition is a key component of the intelligent transportation system. It has broad application prospects in the fields of traffic guidance, assisted driving system, and road monitoring, and it can provide important clues and evidence for public security cases and traffic accident investigation [4,5]. The current target recognition methods mainly include statistical pattern recognition method, template matching recognition method, model recognition method, neural network recognition method, fuzzy set recognition method, and edge detection method [5,6,7]. The edge detection method based on Canny operator is used in this paper. It has a small amount of calculation and high recognition accuracy, and can effectively remove noise and improve recognition reliability [8]. Moreover, its compatibility with NI myRIO is better.

At present, the recognition of vehicle identity still relies mainly on license plate information [9]. The license plate recognition system has become the key to the development of smart cities such as vehicle management, stolen vehicle surveys, and traffic monitoring. When a deck car appears, or the license plate is blocked, vehicle recognition relying solely on the license plate will be ineffective [10]. Therefore, vehicle color and logo are added to vehicle identity recognition. The vehicle recognition method adopted in this paper is based on license plate and supplemented by vehicle color and vehicle logo information, which makes up for the deficiencies in the traditional license plate recognition and greatly improves the accuracy and reliability of the vehicle identity recognition. Thereby, it further improves the reliability of the intelligent transportation system.

In general, color recognition is mainly divided into two steps: (1) color feature extraction and (2) feature classification and matching. Color feature extraction refers to that the color information of the image is statistically analyzed, and is expressed by the feature vector. Feature classification and matching refers to that based on the expression of the given image color, the color category of the image is predicted by a classification or matching algorithm, such as nearest neighbor search, neural network, and support vector machine. From the earliest proposal of using color histogram as a feature to match the existing library, to the proposal of using color invariant moment to describe the color information of the whole object, and then to the proposal of extracting the color feature from the local region of the object to replace the global region, color classification and matching technology has been gradually improved and matured [11]. The vehicle color recognition method based on NI Vision Assistant color classification function is adopted in this paper, which is intuitive, efficient, easy to implement, and has good recognition accuracy.

At present, there are a few researches on the vehicle logo recognition technique, which is a new research direction of vehicle recognition technology. The vehicle logo recognition technique can be widely used in the field of automated safety management, such as criminal vehicle search, traffic accident handling, and illegal vehicle monitoring. However, it is still difficult to develop and implement a complete vehicle logo recognition technique due to the unfixed position, different sizes and various types of the vehicle logo, and the different external environmental conditions. A vehicle logo recognition algorithm based on convolutional neural network is proposed in [12]. The algorithm consists of two parts: knowledge base and inference engine. Its recognition accuracy is good, but the recognition speed is slow and cannot correctly depict the contour features when dealing with the tilted vehicle logo, which has certain influence on recognition. A vehicle logo recognition method based on HOG (Histogram of Oriented Gradient) and PCA (Principal Component Analysis) is proposed in [13]. It uses HOG features in combination with a multiclass SVM (Support Vector Machine) to deal with logo recognition, and the accurate recognition results are obtained. The classification function of KNN (K-nearest neighbor) algorithm based on machine vision is used for training and recognition in this paper. Its recognition speed is fast, the accuracy is high, and a variety of simple vehicle logo can be recognized at the same time.

With the improvement of computer performance and the development of computer vision technology, the research on the license plate recognition technique has gradually developed towards a systematic direction. License plate recognition is an image processing technological solution that captures images of license plates of vehicles, and it segments the characters from the plate area by detecting and extracting the license plate, and then, it displays the license plate information by using feature extraction of the character recognition technique [14]. An improved technique of OCR (Optical Character Recognition)-based license plate recognition using neural network trained dataset of object features is proposed and is compared with the existing methods for improving accuracy in [15]. The license plate recognition scheme based on OCR character recognition algorithm in NI Vision Assistant is adopted in this paper, which realizes the correct recognition of license plates with white characters on a blue background with a length of 8 characters. In the complex environment background, the results of the license plate position recognition and extraction are ideal, the accuracy of character recognition is high, and the recognition effect to the license plate with a large tilting angle is also good.

The purpose of this paper is to design a mobile platform for intelligent transportation systems to achieve vehicle target detection and vehicle information acquisition. NI myRIO is used as the hardware platform and LabVIEW is used as the software platform in this mobile platform. The information of vehicle target is acquired by an industrial camera, and the target detection of the vehicle and the extraction of the vehicle information are simultaneously performed. Finally, the detection result and the acquired information are outputted and displayed.

## 2. Materials and Methods

**Figure 1:**
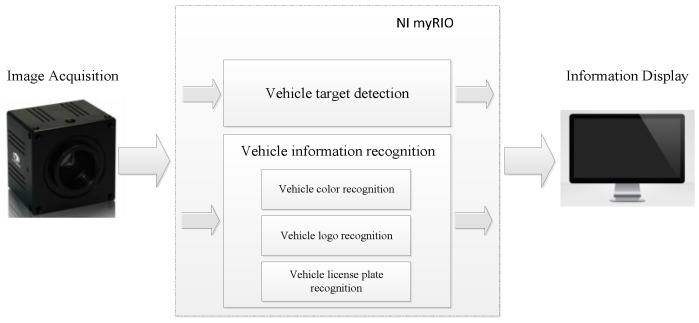
Overall design of vehicle target detection and information extraction system.

The overall flow of vehicle detection and information extraction based on NI myRIO can be observed from the system block diagram in Figure 1. The image input used DH-HV3151UC industrial camera and the main controller was myRIO-s1900 of National Instruments. The DH-HV3151UC industrial camera was a color area array CMOS image sensor with a resolution of 3.1 million pixels, supporting continuous acquisition, external trigger, and soft trigger acquisition. It had a standard USB 2.0 interface that supported hot swap, a DirectX compatible device driver, and a software interface that could be directly recognized by HALCON, LabVIEW, and other software. The core chip of NI myRIO was Xilinx Zynq, which integrated a 667 MHz dual-core ARM Cortex-A9 and an FPGA with 28k logic units, 80 DSP slices, and 16 DMA channels, and an integrated USB host for connecting external devices.

Specifically, the edge detection method was used to implement vehicle target detection, the color classification function was used to implement vehicle color recognition, the pattern recognition method was used to implement vehicle logo recognition, and the OCR character recognition algorithm was used to implement vehicle license plate recognition in real time on NI myRIO. Real-time image data acquisition and processing were realized through the LabVIEW software platform, and the output data were displayed on PC or mobile devices.

## 3. Vehicle Target Detection Scheme and Implementation

### 3.1. Edge Detection Method

The edge of the image refers to the part of the image where the brightness of the local area changes significantly in the digital image. The edge mainly exists between the target and the target, and between the target and the background. It is an important basis for image analysis such as image texture feature extraction and shape feature extraction. Most of the image information is concentrated in the edge of the image. The determination and extraction of the image edge are very important for recognition and analysis of the entire image scene, and they are also an important feature for the image segmentation. Edge detection is mainly the measurement, detection, and localization of grayscale changes of the image. The edge detection is used to detect the vehicle target in this paper. Its advantage is low computational load and high recognition accuracy, and it can be well adapted to NI myRIO environment.

The common edge detection operators in NI Vision Assistant include Laplacian operator, Diff operator, Sobel operator, Roberts operator, and Canny operator, and each of them has a different algorithm. Canny edge detection uses the first-order differentiation of the Gaussian function, which can balance the noise suppression and edge detection process well. Therefore, Canny operator is used to extract the image edge in this paper in order to obtain better edge detection performance.

The two-dimensional Gaussian function is:

(1) $$G\left(x,y\right)=\frac{1}{2\pi \sigma^{2}}exp\left(-\frac{x^{2}+y^{2}}{2\sigma^{2}}\right).$$

In order to improve the operation speed, the convolution ∇G can be decomposed into two one-dimensional row and column filters:

(2) $$\frac{\partial G}{\partial x}=\mathrm{kxexp}\left(-\frac{x^{2}}{2\sigma^{2}}\right)\exp\left(-\frac{y^{2}}{2\sigma^{2}}\right)=h_{1}\left(x\right)h_{2}\left(y\right),$$

(3) $$\frac{\partial G}{\partial y}=kyexp\left(-\frac{y^{2}}{2\sigma^{2}}\right)exp\left(-\frac{x^{2}}{2\sigma^{2}}\right)=h_{1}\left(y\right)h_{2}\left(x\right),$$

where $h_{1}\left(x\right)=\sqrt{k}xexp\left(-\frac{x^{2}}{2\sigma^{2}}\right)$, $h_{1}\left(y\right)=\sqrt{k}yexp\left(-\frac{y^{2}}{2\sigma^{2}}\right)$, $h_{2}\left(x\right)=\sqrt{k}exp\left(-\frac{x^{2}}{2\sigma^{2}}\right)$, $h_{2}\left(y\right)=\sqrt{k}exp\left(-\frac{y^{2}}{2\sigma^{2}}\right)$, and *k* is a constant.

Then, calculate the convolution of Equation (2) and f(x,y) of the image, and Equation (3) and f(x,y) of the image, respectively.

(4) $$P_{x}=\frac{\partial G\left(x,y,\sigma \right)}{\partial x}*f\left(x,y\right),$$

(5) $$P_{y}=\frac{\partial G\left(x,y,\sigma \right)}{\partial y}*f\left(x,y\right).$$

Then, the edge strength *A* and the direction *α* can be obtained, as shown in Equations (6) and (7).

(6) $$A\left(i,j\right)=\sqrt{P_{x}^{2}\left(i,j\right)+P_{y}^{2}\left(i,j\right)},$$

(7) $$\alpha \left(i,j\right)=arctan\frac{P_{y}\left(i,j\right)}{P_{x}\left(i,j\right)}.$$

### 3.2. Vehicle Target Recognition Method

**Figure 2:**
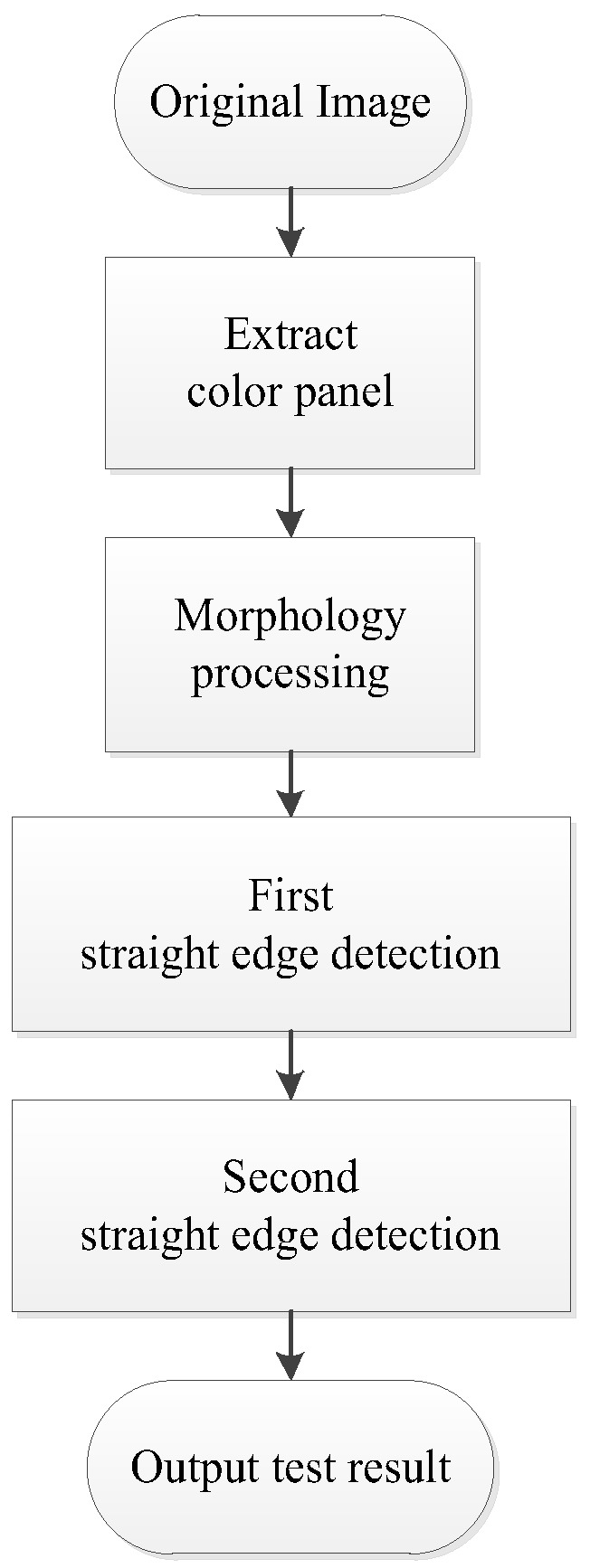
Flowchart of vehicle target recognition scheme.

The vehicle target recognition algorithm in this paper mainly includes image graying processing, morphology processing and two edge detection based on Canny operator. The flowchart of vehicle target recognition scheme is shown in Figure 2.

**Figure 3:**
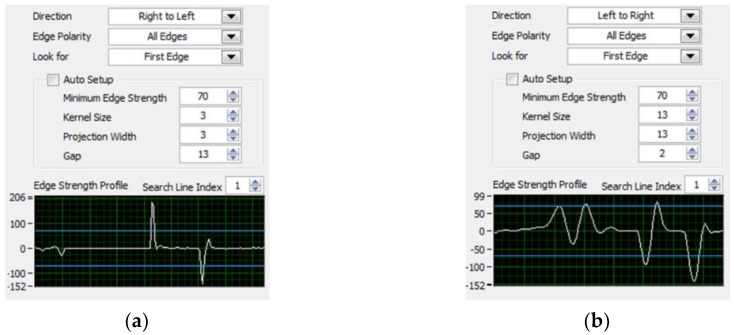
Edge detection parameter settings: (**a**) from right to left and (**b**) from left to right.

**Figure 4:**
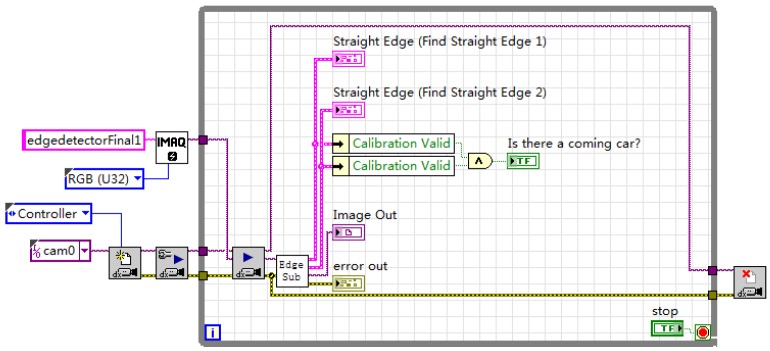
Program block diagram of vehicle target recognition.

The specific steps are as follows: First, extract the luminance plane and grayscale the image. Morphology processing uses 3 × 3 corrosion of the regular octagon to make one iteration. Then, make the edge detection twice, which is from right to left and left to right. If both sides of the vehicle are detected, it is regarded as the vehicle target detected. The edge detection parameter settings are shown in Figure 3, the vehicle target recognition program block diagram is shown in Figure 4.

## 4. Vehicle Color Recognition Scheme and Implementation

**Figure 5:**
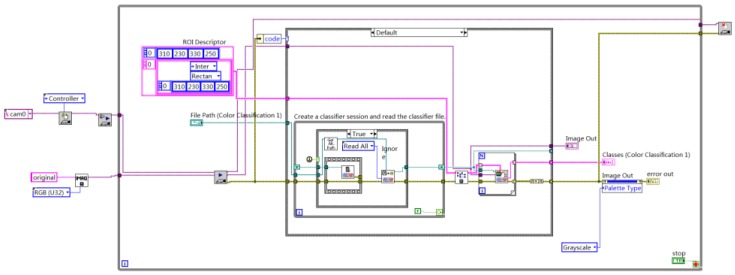
Program block diagram of color recognition.

**Figure 6:**
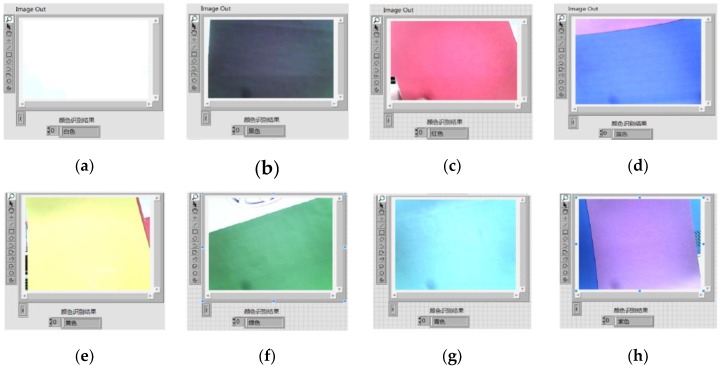
Color recognition results: (**a**) white, (**b**) black, (**c**) red, (**d**) blue, (**e**) yellow, (**f**) green, (**g**) cyan, and (**h**) purple.

The vehicle color recognition method based on the color classification function of NI Vision Assistant is used in this paper. First, create a color sample class and color samples using NI Vision Assistant. The number of color samples should not be too small or too large, so each group of color classes adds 5–10 samples under different light. The sensitivity color recognition based on brightness is adopted for sample recognition. The nearest neighbor training algorithm is used for sample training. The color recognition process outputs each color recognition score and displays the result with the highest score, thereby realizing the recognition of eight colors of red, yellow, green, cyan, blue, purple, black, and white. The program block diagram of color recognition is shown in Figure 5. The eight color recognition results are shown in Figure 6.

## 5. Vehicle Logo Recognition Scheme and Implementation

### 5.1. Classification Algorithm Introduction

The KNN algorithm is called K-nearest neighbor, and it is a no parent statistical method proposed by Cover and Hart in 1968 for classification and regression. Classification is made by measuring the distance between different eigenvalues in KNN. The specific method is that if the majority of the K most similar samples of a sample, which is also the nearest, in a feature space belong to a certain category, that sample also belongs to this category, where K is usually an integer not greater than 20. In the KNN algorithm, the selected neighbors are all objects that have been correctly classified.

When the data and labels in the training set are known, enter the test data. Compare the features of the test data with the features corresponding to the training set to find the top K data most similar to the test data, and the category that the test data corresponding to is the one with the most occurrences among the K data. The algorithm steps are:

(1) Calculate the distance between the test data and each training data;
(2) Sorting according to the increasing relationship of distances;
(3) Select the K points with the small distance;
(4) Determine the occurrence frequency of the category of the first K points;
(5) Return the category with the highest frequency among the top K points as the prediction category of the test data.

In the KNN algorithm, there are three commonly used distances, namely, Manhattan distance, Euclidean distance, and Minkowski distance.

Let the feature space X be an n-dimensional real number vector space. $x_{i},x_{j}\in X,x_{i}={\left(x_{i}{}^{\left(1\right)},x_{i}{}^{\left(2\right)},\ldots ,x_{i}{}^{\left(n\right)}\right)}^{T}$, $x_{j}={\left(x_{j}{}^{\left(1\right)},x_{j}{}^{\left(2\right)},\ldots ,x_{j}{}^{\left(n\right)}\right)}^{T}$, and the distance $L_{p}$ of $x_{i},x_{j}$ is defined as

(8) $$L_{p}\left(x_{i},x_{j}\right)={\left(\sum_{l=1}^{n}{\left|x_{i}{}^{\left(l\right)}-x_{j}{}^{\left(l\right)}\right|}^{p}\right)}^{\frac{1}{p}},$$

here *p* ≥ 1. 

When *p* = 1, it is called Manhattan distance, and the formula is:

(9) $$L_{1}\left(x_{i},x_{j}\right)=\sum_{l=1}^{n}\left|x_{i}{}^{\left(l\right)}-x_{j}{}^{\left(l\right)}\right|.$$

When *p* = 2, it is called Euclidean distance, and the formula is:

(10) $$L_{2}\left(x_{i},x_{j}\right)={\left(\sum_{l=1}^{n}{\left|x_{i}{}^{\left(l\right)}-x_{j}{}^{\left(l\right)}\right|}^{2}\right)}^{\frac{1}{2}}.$$

When *p* = ∞, it is the maximum value of each coordinate distance, and the formula is:

(11) $$L_{\infty }\left(x_{i},x_{j}\right)=max_{l}\left|x_{i}{}^{\left(l\right)}-x_{j}{}^{\left(l\right)}\right|.$$

### 5.2. Vehicle Logo Recognition Scheme

**Figure 7:**
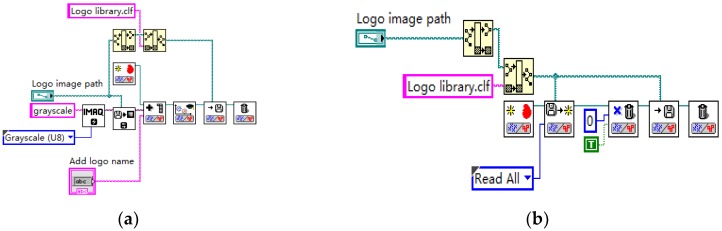
Program block diagram. (**a**) creation of library files and (**b**) deletion of the library files.

**Figure 8:**
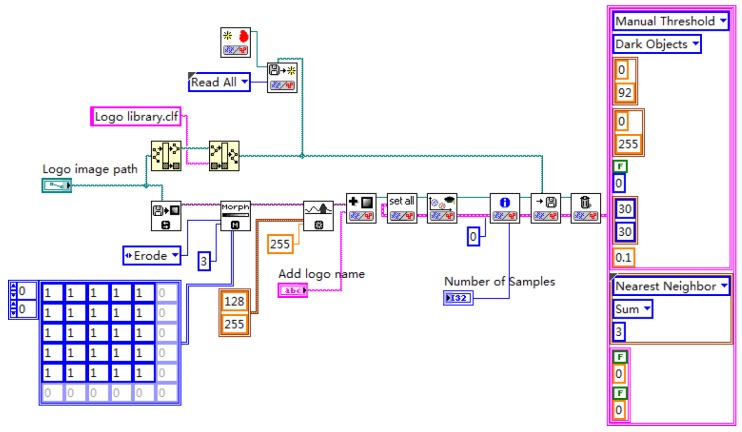
Program block diagram of addition of the sample.

The vehicle logo recognition based on pattern matching is adopted in this paper, which is divided into three parts: the production of library files, the preprocessing of images, and the recognition display of vehicle logos. The production library file includes the creation of the library file, addition and deletion of the vehicle logo sample, as shown in Figure 7 and Figure 8. The vehicle logo images in different lights are added to the sample library trained by a classification function based on the KNN algorithm.

**Figure 9:**
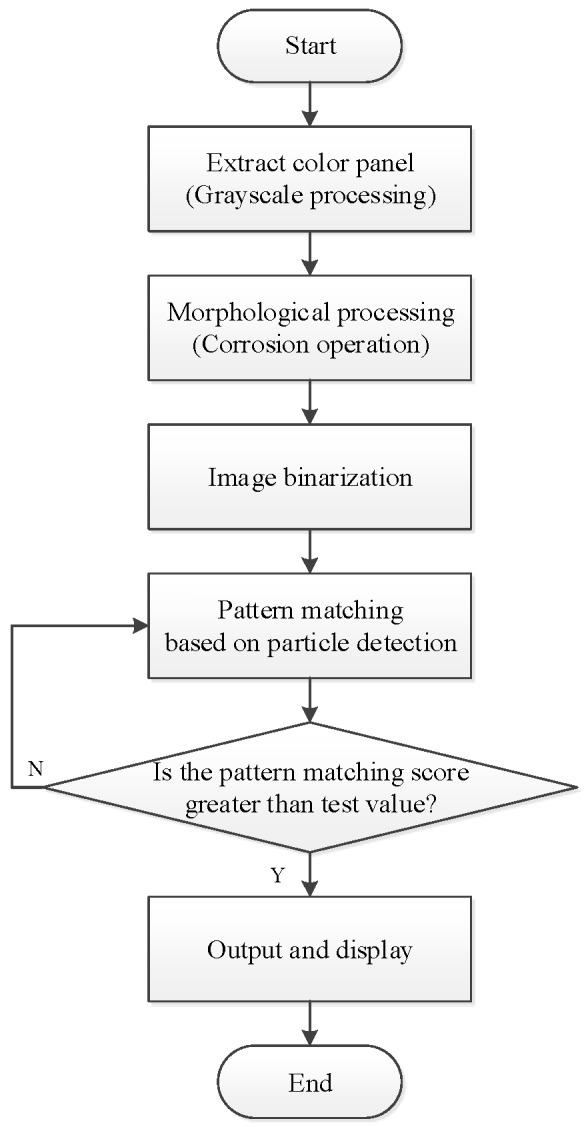
Process of preprocessing and vehicle logo recognition.

**Figure 10:**
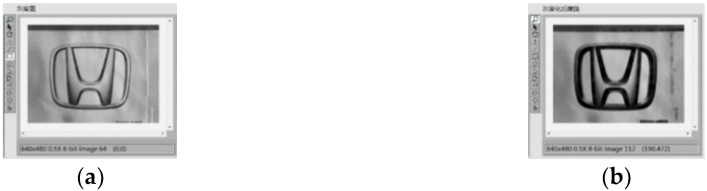
Corrosion operation: (**a**) before and (**b**) after.

**Figure 11:**
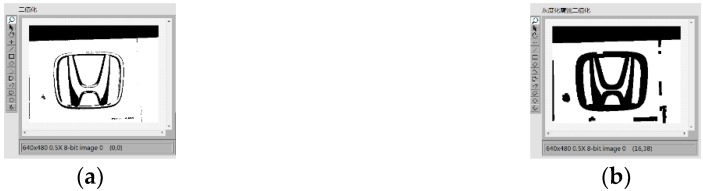
Binarization: (**a**) before and (**b**) after.

**Figure 12:**
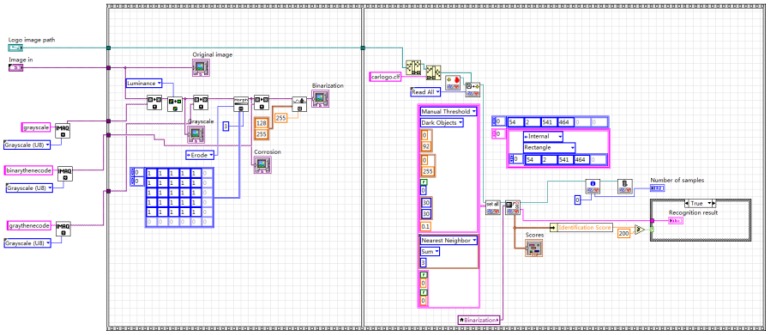
Overall program block diagram of the vehicle logo recognition.

**Figure 13:**
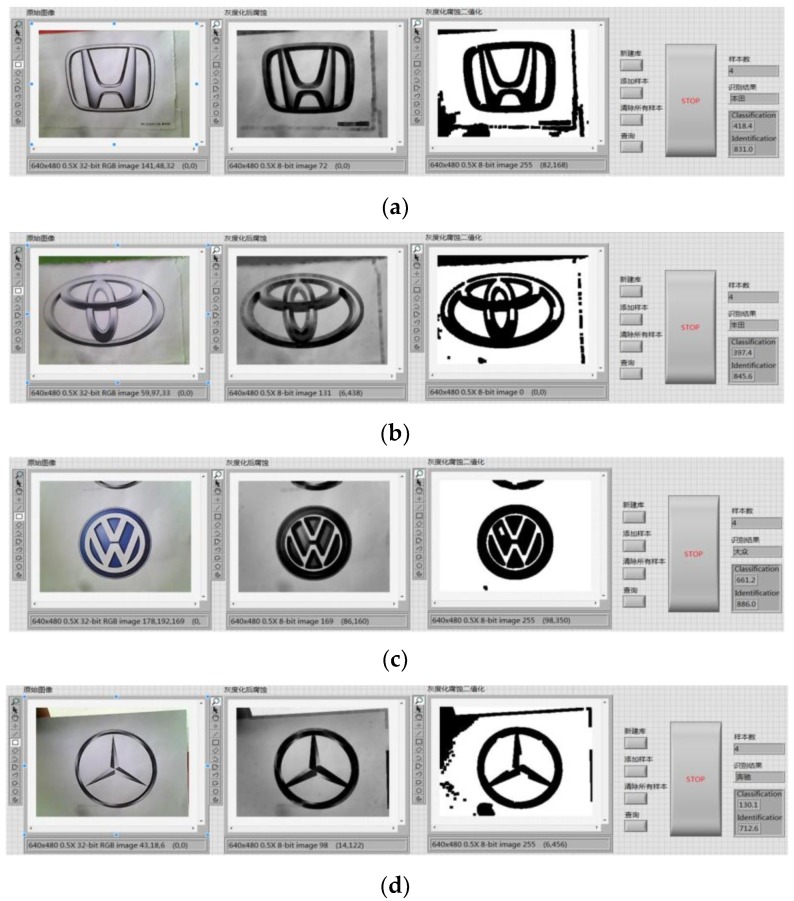
Vehicle logo recognition results: (**a**) Honda, (**b**) Toyota, (**c**) Volkswagen, and (**d**) Mercedes-Benz.

Image preprocessing uses grayscale corrosion and then binarization. The classification function in pattern matching is used to compare important features with the features of the known object category. The preprocessing and vehicle logo recognition process is shown in Figure 9. First, the acquired image is preprocessed and the brightness plane is extracted to convert the 32-bit color image into an 8-bit grayscale image. Then, carry out corrosion operations to the image by using 5 5 corrosion of the square and make three iterations, as shown in Figure 10. Then, carry out the image binarization. Set the gray value of the pixel with the gray value from 0 to 127 to 0, and the rest to 255, as shown in Figure 11. Pattern matching and vehicle logo recognition are performed by the classification function and the matching result is judged. After multiple tests, when the test value is 700, the identification requirements can be met. So, the result of the score greater than or equal to test value is output. The recognition of Honda, Toyota, Volkswagen, Mercedes-Benz, and other vehicle logos is realized. The overall program block diagram of the vehicle logo recognition is shown in Figure 12, and the recognition results are shown in Figure 13.

## 6. License Plate Recognition Scheme and Implementation

### 6.1. OCR Character Recognition

The OCR (Optical Character Recognition) technique refers to that an electronic device, such as a scanner or digital camera, checks the character printed on paper, determines its shape by detecting dark and bright patterns, and then translates the shape into computer characters by character recognition.

### 6.2. License Plate Recognition Scheme

**Figure 14:**
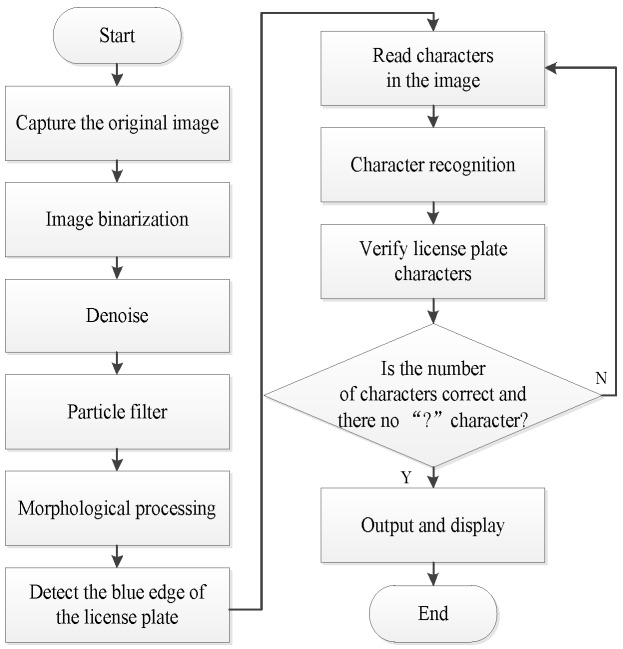
Design flow chart of license plate recognition.

**Figure 15:**
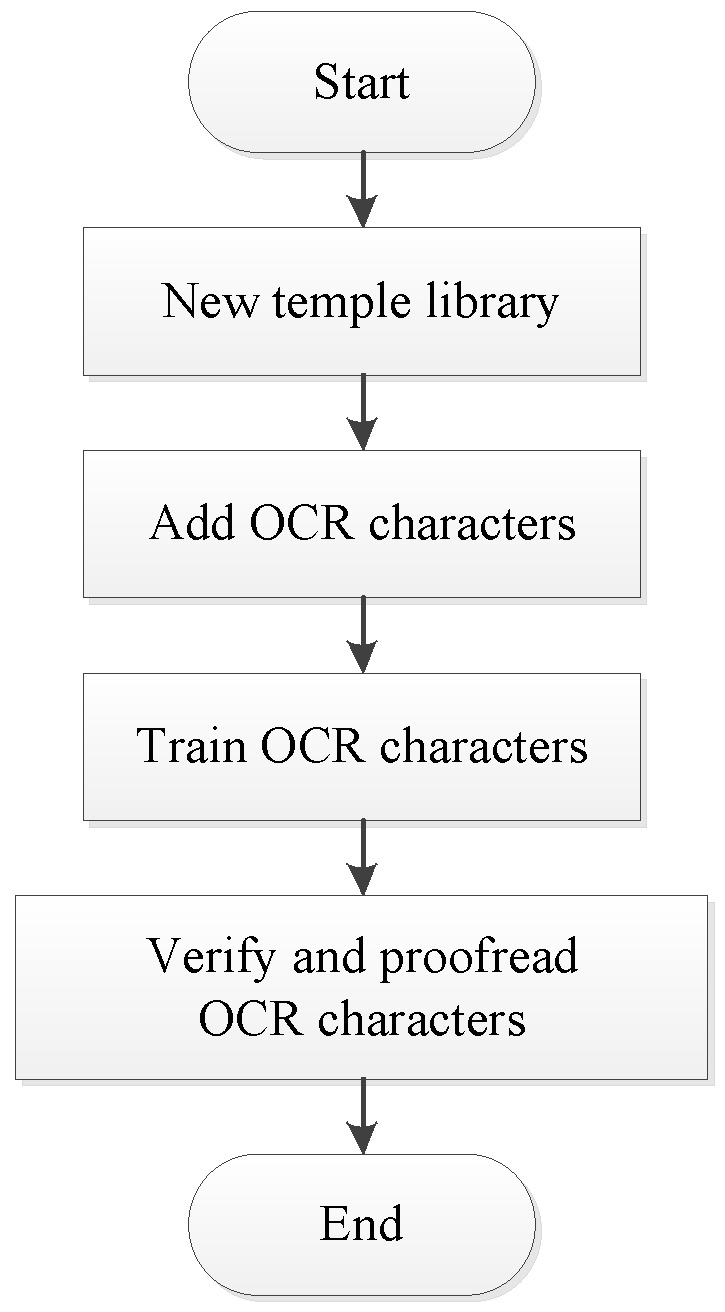
Design flow chart of Optical Character Recognition (OCR) character recognition.

The license plate recognition scheme mainly includes image acquisition, image preprocessing, license plate position extraction, character segmentation, and character recognition. The license plate recognition scheme based on OCR character recognition algorithm in NI Vision Assistant is used in this design. The design flow chart of license plate recognition is shown in Figure 14, and the design flow chart of OCR character recognition is shown in Figure 15.

**Figure 16:**
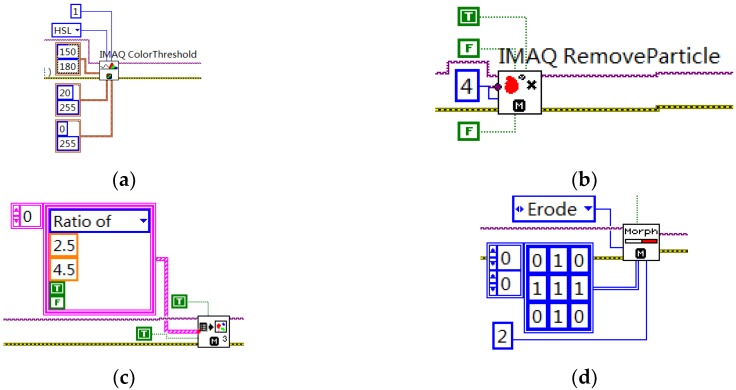
Program block diagram: (**a**) binarization, (**b**) denoising, (**c**) particle filter, and (**d**) corrosion operation.

Image preprocessing: It includes binarization, removal of noise interference, particle filtering and morphological transformation processing operations of dynamically acquired images. First, the color image is binarized. The value of the pixels in the HSL(Hue Saturation Lightness) plane with a tone value of 150–180 and saturation value of 20–180 are replaced with white, and the others are replaced with black. Then the image is denoised. The IMAQ RemoveParticle VI function is used to carry out particle detection in connection mode 8. The number of corrosion operations is 8, and the shape is octagonal. Then, the particle is filtered to remove the interference particles. Finally, a 3 3 octagon corrosion operation is carried out to the image, and the number of iterations is 2. The program block diagram is shown in Figure 16.

**Figure 17:**
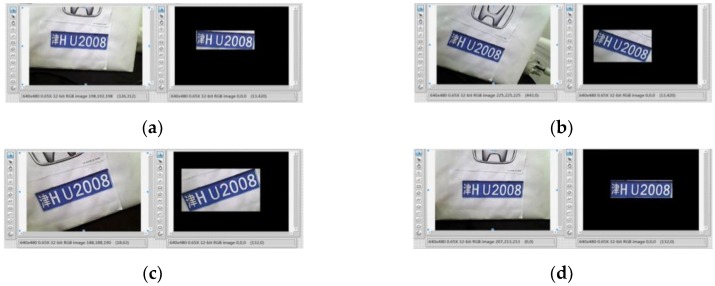
License plate positioning results: (**a**) Position 1, (**b**) Position 2, (**c**) Position 3, and (**d**) Position 4.

License plate position extraction: The background of vehicle images is often complicated in the natural environment, so how to determine the license plate position and extract the license plate position information is the key to the entire license plate recognition. In this paper, the license plate position is determined by detecting the blue edge of the license plate and the license plate is segmented from the image. The IMAQ Particle Analysis VI function is used to carry out particle analysis to the preprocessed image, and the boundary pixel value of the license plate area is extracted. The license plate positioning results are shown in Figure 17.

**Figure 18:**
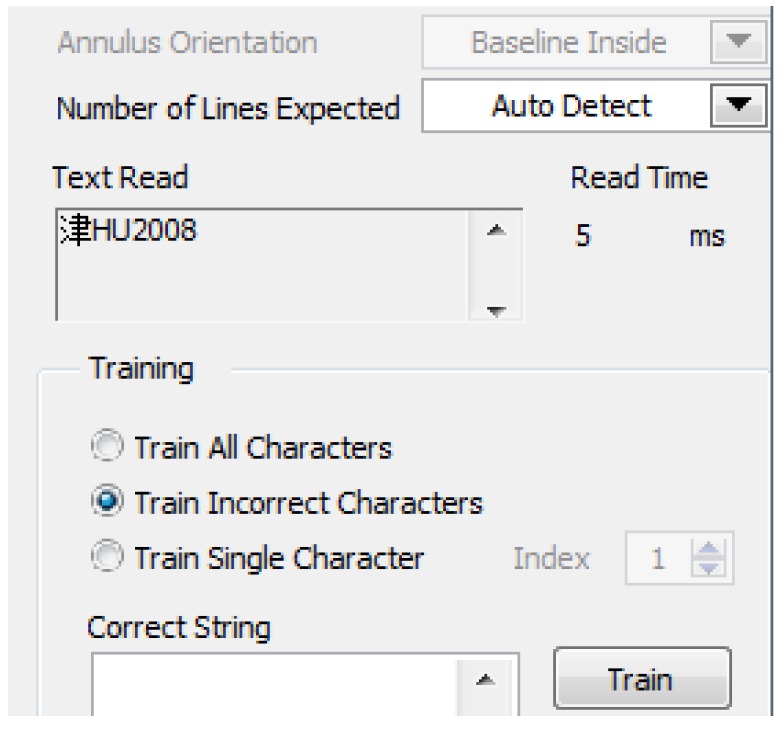
Character training interface.

**Figure 19:**
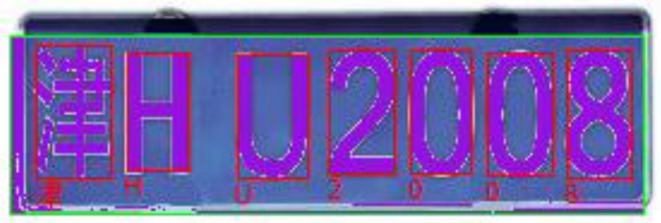
Character training result.

**Figure 20:**
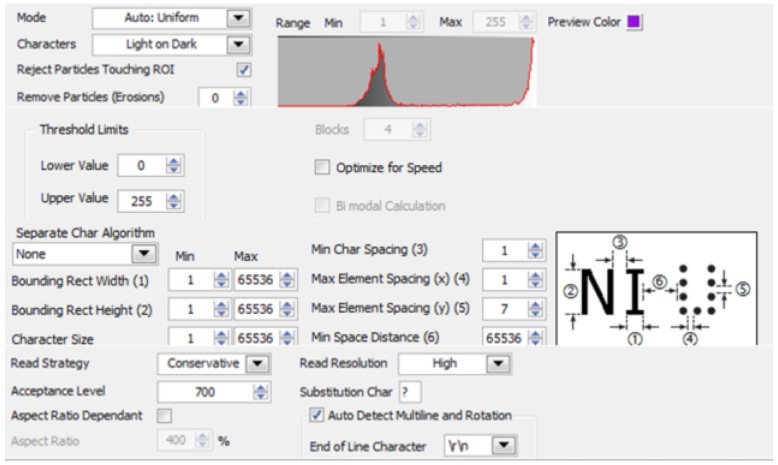
Character recognition parameter settings.

**Figure 21:**
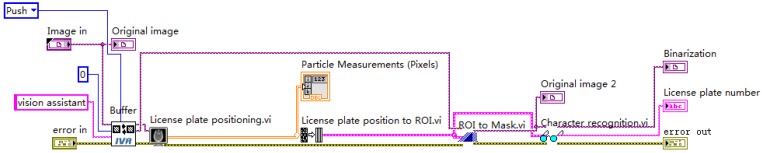
Main program block diagram of license plate recognition.

**Figure 22:**
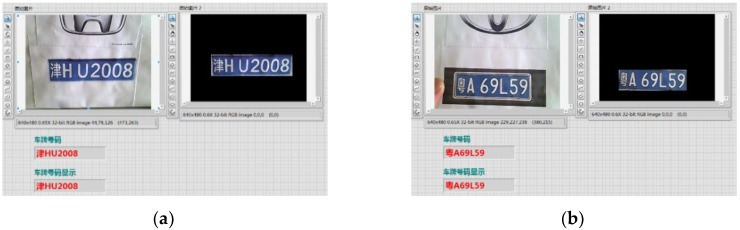
License plate recognition results: (**a**) License Plate 1 and (**b**) License Plate 2.

Character recognition and verification: The OCR character recognition algorithm is used in character segmentation and character recognition in this paper. First, train the OCR character library, and save the trained character template as a file. The character training interface is shown in Figure 18, and the training result is shown in Figure 19. When the character is recognized, the characters of the license plate is compared with that in the character template. The result closest to the character template is output, and the license plate information is displayed. The character recognition parameter settings are shown in Figure 20. In the verification, the errors and the recognition results with “?” are eliminated, realizing the correct recognition of license plates with white characters on a blue background with a length of 8 characters. The results of the license plate position recognition and extraction are ideal and the accuracy of the character recognition is also high. The main program block diagram of license plate recognition is shown in Figure 21, and the recognition results are shown in Figure 22.

## 7. System Integration and Performance Analysis

**Figure 23:**
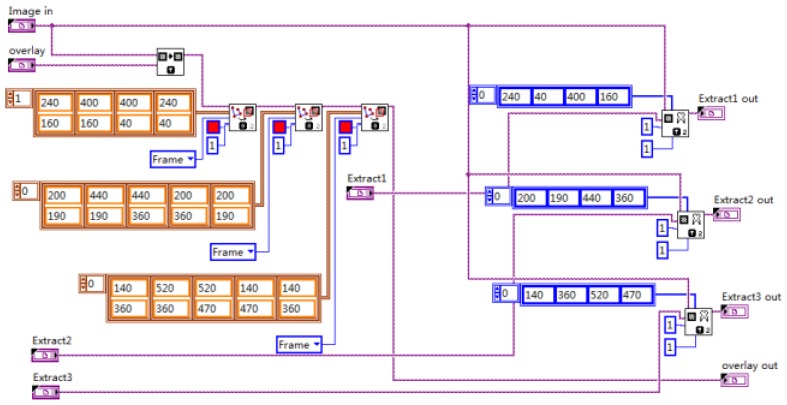
Program block diagram of image segmentation and extraction.

**Figure 24:**
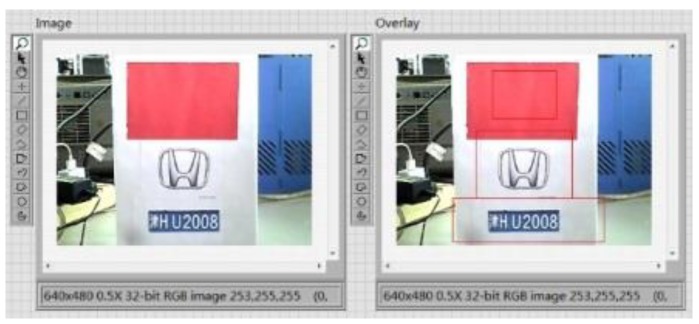
Image segmentation result.

**Figure 25:**
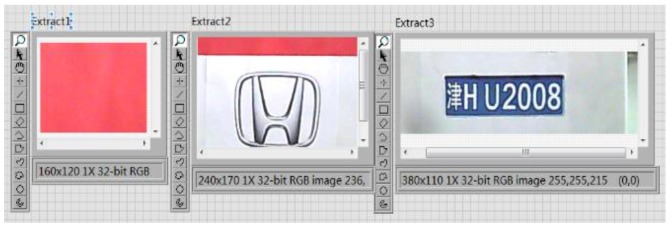
Image extraction result.

After each module of the vehicle target detection and information extraction system is completed, the whole system is integrated. The whole image is partitioned into three regions and marked with red lines. The program block diagram of image segmentation and extraction is shown in Figure 23, and the results are shown in Figure 24 and Figure 25.

**Figure 26:**
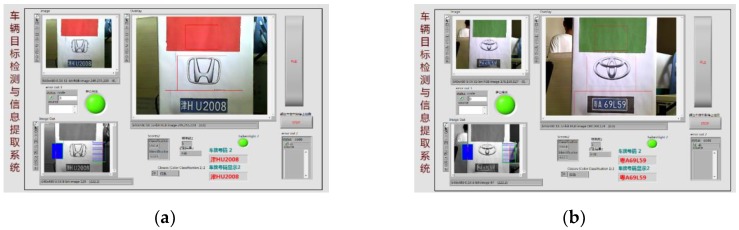
Overall system recognition results: (**a**) Vehicle 1 and (**b**) Vehicle 2.

**Figure 27:**
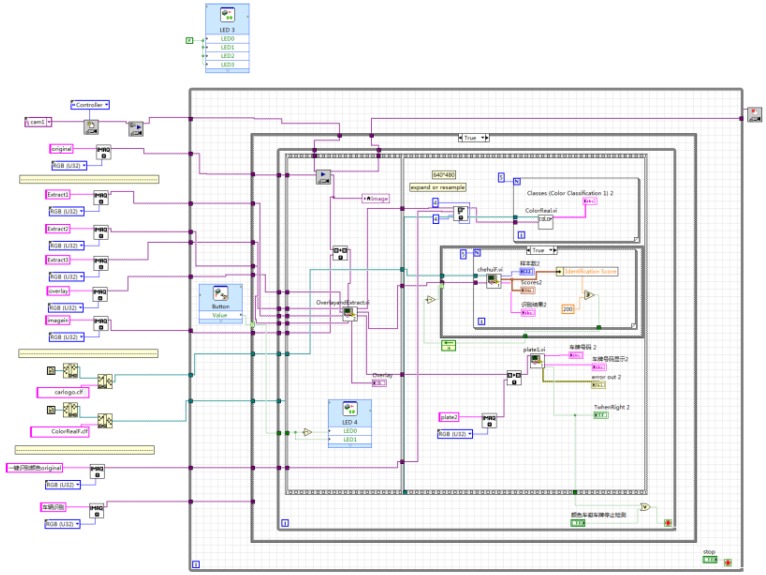
Overall system program block diagram.

The user-defined button of NI myRIO is used to control the program to run in the system. When the button is released, the vehicle target detection is carried out. When the button is pressed, the vehicle information extraction and recognition function is carried out to identify the color, logo, and license plate information of the vehicle, and the real-time images and recognition results are displayed on the front panel. LED0~LED2 are used to display the real-time status of the system program. When the vehicle target detection program is running, LED0 lights up. When the vehicle information extraction and recognition program is running, LED1 lights up. When there is a car coming, LED2 lights up, whereas LED2 goes out when there is no car coming. The recognition result is shown in Figure 26, and the overall system program block diagram is shown in Figure 27.

## 8. Conclusions

The vehicle detection and information extraction system in this paper uses the NI myRIO hardware platform to realize vehicle target detection based on edge detection, vehicle color recognition based on color classification function, vehicle logo recognition based on pattern matching, and license plate recognition based on OCR character recognition algorithm. The system can detect the vehicle target in real time, and determine the vehicle identity according to the color, logo, and license plate information of the vehicle. The recognition speed and accuracy are nice. The development cycle is shortened and the scalability is good by using the visual component LabVIEW programming environment.

The system will be expanded and optimized in the future for more vehicle target processing tasks, including target object segmentation, target object tracking, deep learning, and so on, to further improve the system’s functions and performance.
